# Malignant ascites enhances migratory and invasive properties of ovarian cancer cells with membrane bound IL-6R *in vitro*

**DOI:** 10.18632/oncotarget.13074

**Published:** 2016-11-04

**Authors:** Soochi Kim, HyeRan Gwak, Hee Seung Kim, Boyun Kim, Danny N. Dhanasekaran, Yong Sang Song

**Affiliations:** ^1^ Interdisciplinary Program in Cancer Biology, College of Medicine, Seoul National University, Seoul, Republic of Korea; ^2^ Cancer Research Institute, Seoul National University, Seoul, Republic of Korea; ^3^ Biomodulation, Department of Agricultural Biotechnology, Seoul National University, Seoul, Republic of Korea; ^4^ Department of Obstetrics and Gynecology, Seoul National University, Seoul, Republic of Korea; ^5^ Nano System Institute, Seoul National University, Seoul, Korea; ^6^ Stephenson Cancer Center, University of Oklahoma Health Sciences Center, USA

**Keywords:** ascites, migration, invasion, IL-6R, ovarian cancer

## Abstract

Transcoelomic route is the most common and the earliest route of metastasis, causing the ascites formation in advanced epithelial ovarian cancer (EOC). We demonstrated that interleukin 6 (IL-6) is enriched in the malignant ascites from patients with ovarian cancer, which enhanced invasive properties of EOC cells. Interestingly, the expression of IL-6R on cell membrane of EOC cells correlated with ascites-induced invasion. Selective knockdown of IL-6R or inhibition with IL-6 neutralizing antibody, suppressed the stimulatory effects of ascites on EOC invasion. Moreover, the ascites treatment induced the phosphorylation of JAK2-STAT3 and use of selective inhibitors of JAK2 and STAT3, blocked the expression of epithelial-mesenchymal transition related proteins in parallel with the suppression of EOC invasion. Thus, IL-6/IL-6R mediated JAK2-STAT3 signaling pathway could be a promising therapeutic target for anticancer therapy in ovarian cancer patients with ascites.

## INTRODUCTION

Epithelial ovarian cancer (EOC) is the most lethal gynecological malignancy due to the frequent relapse and chemoresistance. Asymptomatic nature of the disease, largely contributes to the diagnosis of patients in advanced stage III/IV, causing a greater than 60% mortality rate within five years [1]. In contrast to other solid tumor, the metastatic pattern of ovarian cancer is unique. The most common and earliest route of metastasis is transcoelomic, which frequently causes the accumulation of fluid in the peritoneal cavity, called ascites [2]. Clinically, the presence of ascites is significantly associated with a decreased quality of life and a poor prognosis [3, 4].

In general, ascites formation provides a favorable environment for tumor cells. The role of ascites in ovarian cancer progression is diverse, including promotion of proliferation, spheroid formation, attenuation of TRAIL-induced apoptosis and enhanced invasive behavior [5–7]. Ascites are composed of both cellular and acellular components, each of which has a distinctive but cumulative role in disease progression. The concentration of bio-active molecules present in ascites varies between patients, according to their disease stage, grade and histological subtypes [8, 9]. Variety of soluble factors including inflammatory cytokines has been demonstrated to individually affect EOC progression through different mechanisms. Of those, high level of IL-6 in the serum and ascites of the cancer patients has been shown to be associated with worse clinical outcomes [10, 11]. The signaling pathway downstream of IL-6, especially JAK-STAT3 pathway is aberrantly activated and is associated with the cancer progression, though this pathway is an essential component of normal development and homeostasis [12].

Ovarian cancer cells in malignant ascites have also been characterized by epithelial mesenchymal transition (EMT), supporting the idea that ascites contains factors mediating the EMT pathway and metastasis [13]. Although previous studies have demonstrated the stimulatory role of ascites in migration and invasion of EOC cells, its exact mechanism has not been determined. Herein we report that human ovarian cancer patient derived ascites comprises pro-inflammatory tumor microenvironment (TME), of which elevated levels of IL-6 increased EOC cell invasion through JAK2-STAT3 signaling in parallel with increased expression of EMT related genes. Furthermore, our data indicated that, the expression of IL-6R on cell membrane of EOC cells is correlated with ascites induced invasion *in vitro*.

## RESULTS

### Ascites promotes migration and invasion of EOC cells

**Figure 1:**
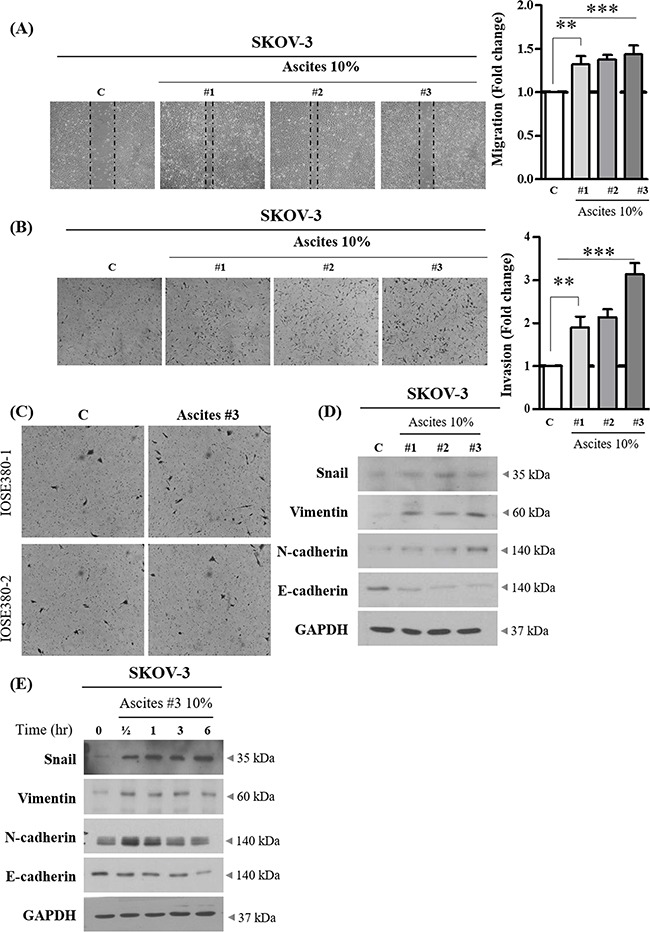
Effect of ovarian cancer patient derived ascites on SKOV-3 cell migration and invasion **A.** SKOV-3 cancer cells were treated with or without 10% ascites. After 24 hr, wound healing ability was verified by measuring wound closed area under a light microscope (magnification x 40). **B.** SKOV-3 cancer cells were seeded into the upper chamber of Matrigel-coated membrane in transwells. Cell invasion were induced with or without 10% ascites. After 24 hr, invaded cells at the bottom of the transwell were stained with 0.5% crystal violet and were counted under a light microscope (magnification x 200). **C.** IOSE380 cells were seeded into the upper chamber of Matrigel-coated membrane in transwells. Cell invasion were induced and counted as above. **D.** SKOV-3 cancer cells were treated with or without 10% ascites. After 24 hr, the expression levels of EMT molecular markers, Snail, Vimentin, N-cadherin and E-cadherin were examined by western blot. GAPDH was used as an internal control. **E.** SKOV-3 cancer cells were treated with or without 10% ascites for 0 – 6 hr. Total cell lysates were extracted and subjected to western blot as above. ** and *** represent *P* < 0.01 and *P* < 0.001, respectively.

To assess the role of ascites in the migration and invasion of ovarian cancer cells, three ascites were randomly selected from women with advanced serous ovarian carcinoma, which is the most commonly encountered ovarian cancer subtype in clinical intervention. SKOV-3 cancer cell line, the most commonly used cellular models of ovarian cancer were used to confirm the effect of ascites on EOC cell migration and invasion. Using *in vitro* migration (wound healing) and invasion (Matrigel-coated transwell) assay, we found all three ascites from ovarian cancer patients increase migration and invasion in SKOV-3 (Figure 1A and 1B). However, this phenomenon was only confined to SKOV-3 ovarian cancer cells, and did not occur in normal immortalized ovarian surface epithelial cells (IOSE380) (Figure 1C). It has been established that cells with mesenchymal phenotype are endowed with enhanced migration and invasive capabilities [14] and EMT-dependent invasion and metastasis programs are strongly responsive to microenvironment changes [15, 16]. Therefore, we determined the effect of ascites on the expression of EMT related proteins. We found all three ascites from ovarian cancer patients reduced the expression of an epithelial marker (E-cadherin), and increased the expression of mesenchymal markers (Snail and Vimentin) (Figure 1D and 1E) and these changes were statistically significant (Supplementary Figure S1A and S1B). Although, the expression of N-cadherin is induced in the first 30 min of ascites treatment and decreased thereafter, ascites treatment decreased overall E-/N-cadherin ratio (Supplementary Figure S1C and S1D).

### High levels of pro-inflammatory cytokines in malignant ascites from patients with ovarian cancer

**Figure 2:**
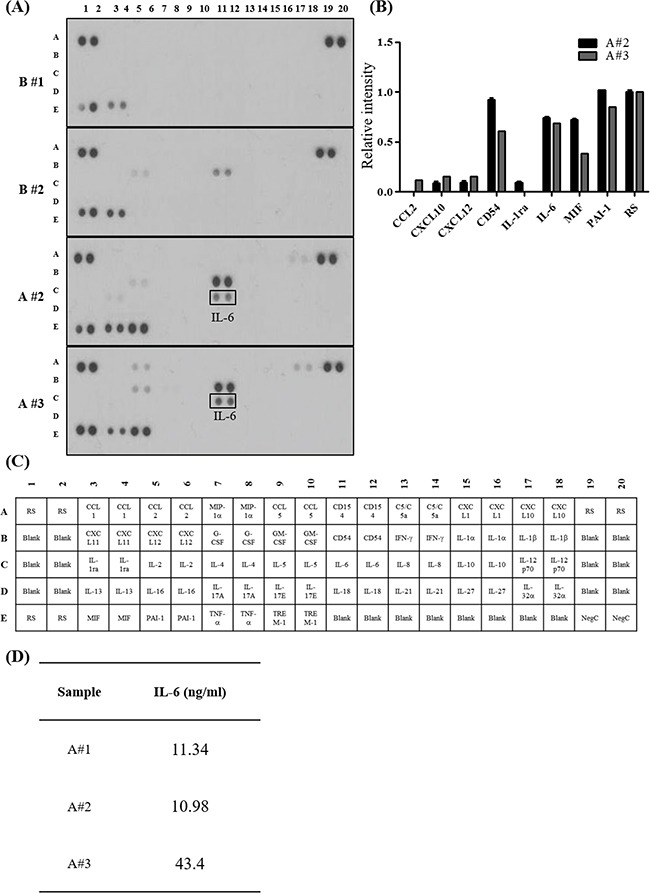
Pro-inflammatory cytokines enriched in ovarian cancer patient derived ascites **A.** Human cytokine array. The array key represents the location of each antibody in duplicate on the membrane. Peritoneal fluid collected from patients with benign condition (**top two panels**) and ovarian cancer patient derived ascites (**bottom two panels**). **B.** Relative intensity of each spot on the membranes were measured and normalized to the reference spot (RS). Representative cytokines enriched in ovarian cancer patient derived ascites are listed on the graph. **C.** Human cytokine array panel. **D.** IL-6 concentration in ovarian cancer patient derived ascites. The concentration of IL-6 was determined by ELISA.

Ascites constitutes a dynamic reservoir of soluble factors, which individually and in a combined fashion may affect tumor cells behavior [17]. To determine the cytokine(s) in ascites that are associated with EMT-dependent invasion of SKOV-3 cells, we evaluated a panel of cytokines using a cytokine array. Using two peritoneal fluids as benign control (Table 1, description of patients), the presence of pro-inflammatory cytokines in ovarian cancer patient derived ascites were compared. From relative comparison, we found IL-6 expression only in ovarian cancer patient derived ascites (Figure 2A and 2B). Then we applied enzyme-linked immunosorbent assay (ELISA), to measure the IL-6 levels. IL-6 was present at high levels (> 3 ng/ml) in all three tested ascites (Figure 2D).

**Table 1:** Description of patients recruited in the study

	Patient ID	Disease	Histopathology	STAGE	Age at diagnosis	Survival	Survival status	Tumor spread
Cancer	A#1	Ovarian cancer	Serous	IV	79	2 years as of 09/06/16	Alive	Ovary, omentum, pelvic lymph node, colon serosa, diaphragm
	A#2	Ovarian cancer	Serous	IIIC	72	2 years as of 27/06/16	Alive	Ovary omentum, pelvic lymph node, colon serosa, appendix, diaphragm
	A#3	Ovarian cancer	Serous	IV	68	1 year 11 months as of 19/07/16	NA	Ovary, omentum, bilateral sub-diaphragmatic, gallbladder, left obturator
Benign	B#1	Para-tubal cyst	NA	NA	76		Alive	
	B#2	Ovarian mucinous cystadenoma	NA	NA	55		Alive	

* NA: Not available

### IL-6 in ascites increase migration and invasion via JAK2-STAT3 signaling

**Figure 3:**
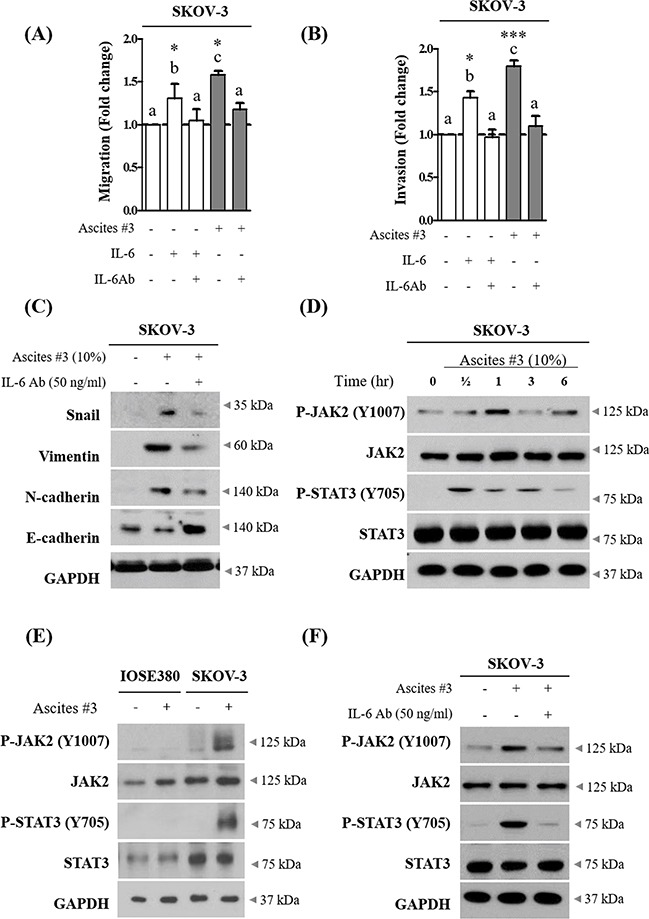
IL-6 in ovarian cancer patient derived ascites increase migration and invasion of SKOV-3 cells via JAK2-STAT3 signaling **A.** SKOV-3 cancer cells were treated with 10% ascites that were either pre-treated with or without 50 ng/ml of IL-6 antibody (IL-6 Ab) for 6 hr. Recombinant IL-6 (10ng/ml) was used as a positive control. After 24 hr, wound healing ability was verified by measuring wound closed area under a light microscope (magnification x40). **B.** SKOV-3 cancer cells were seeded into the upper chamber of Matrigel-coated membrane in transwells. Cell invasion were induced by ascites as above. After 24 hr, invaded cells at the bottom of the transwell were stained with 0.5% crystal violet and counted under a light microscope (magnification x200). **C.** SKOV-3 cancer cells were treated with IL-6 Ab as above. After 24 hr, the expression of Snail, Vimentin, N-cadherin and E-cadherin were examined by western blot. GAPDH was used as an internal control. **D.** SKOV-3 cancer cells were treated with or without 10% ascites for 0 - 6 hr. The expression of p-JAK2 (Y1007), JAK2, p-STAT3 (Y705) and STAT3 were examined by western blot. GAPDH was used as an internal control. **E.** IOSE380 and SKOV-3 cells were treated with or without 10% ascites. After 0.5 hr, the expression of p-JAK2 (Y1007), JAK2, p-STAT3 (Y705) and STAT3 were examind by western blot. GAPDH was used as an internal control. **F.** SKOV-3 cancer cells were treated with IL-6 Ab as above. The expression of p-JAK2, JAK2, p-STAT3 and STAT3 were examined by western blot. GAPDH was used as an internal control. * and *** represent *P* < 0.05 and *P* < 0.001, respectively.

**Figure 4:**
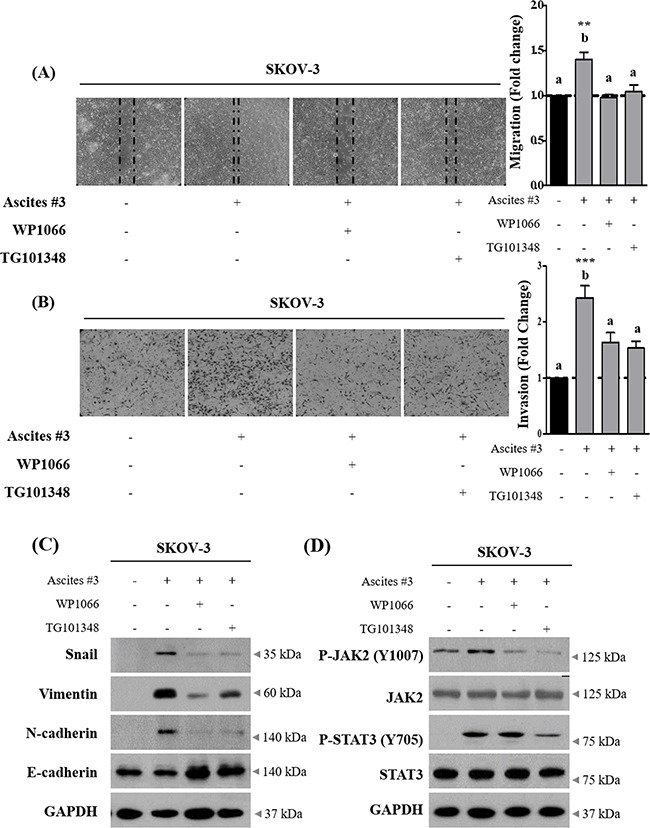
Inhibition of JAK2-STAT3 signaling suppress ascites-induced migration and invasion in SKOV-3 cells **A.** SKOV-3 cancer cells were treated with 10% ascites, with or without JAK2 and STAT3 inhibitors, WP1066 and TG101348. After 24 hr, wound healing ability was verified by measuring wound closed area under a light microscope (magnification x40). **B.** SKOV-3 cancer cells were seeded into the upper chamber of Matrigel-coated membrane in transwells. Cell invasion were induced by ascites with or without JAK2 and STAT3 inhibitors. After 24 hr, invaded cells at the bottom of the transwell were stained with 0.5% crystal violet and counted under a light microscope (magnification x200). **C.** SKOV-3 cancer cells were treated with JAK2 and STAT3 inhibitors as above. After 24 hr, the expression of Snail, Vimentin, N-cadherin and E-cadherin were examined by western blot. GAPDH was used as an internal control. **D.** SKOV-3 cancer cells were treated as above. The expression of p-JAK2 (Y1007), JAK2, p-STAT3 (Y705) and STAT3 were examined by western blot. GAPDH was used as an internal control. ** and *** represent *P* < 0.01 and *P* < 0.001, respectively.

To investigate whether IL-6 enriched in ascites increased SKOV-3 cell migration and invasion, we applied neutralization approach. Ascites were pre-treated with neutralizing IL-6 antibody for 6 hr. This process suppressed the enhanced migration and invasion in SKOV-3 cells (Figure 3A and 3B). Importantly, we also found that neutralization of IL-6 in ascites suppressed the effect of ascites on the expression of EMT related proteins (Figure 3C and Supplementary Figure S3A). Moreover, ascites treatment did not affect IL-6 autocrine expression in SKOV-3 cells (Supplementary Figure S2A and S2B). Perturbed JAK2-STAT3 signaling pathway is implicated in a range of cancers and influences various cellular processes including invasion [18, 19]. To investigate this point, we first examined the effect of ascites on tyrosine phosphorylation of JAK2 and STAT3 in SKOV-3 cells. As expected, we found that ascites treatment induced both JAK2 and STAT3 phosphorylation on Tyr1007 and Tyr705, respectively. STAT3 was rapidly phosphorylated upon ascites treatment, reaching the highest level at the 30 min time point, and declined rapidly (Figure 3D and Supplementary Figure S3B). However, this phenomenon was only confined to ovarian cancer cells, SKOV-3 cells, and did not occur in normal IOSE380 cells (Figure 3E and Supplementary Figure S3C). Moreover, pre-treatment with neutralizing IL-6 antibody as above, suppressed the ascites induced JAK2 and STAT3 phosphorylation (Figure 3F and Supplementary Figure S3D). To further validate the necessity of JAK2-STAT3 in ascites enhanced invasion in SKOV-3 cells, we used WP1066 (an inhibitor of JAK2 and STAT3, 2 μM) and TG101348 (an inhibitor for JAK2, 1 μM) to expectably suppress the JAK2 and STAT3 signaling activity. Both inhibitors significantly suppressed the enhanced migration and invasion in SKOV-3 cells (Figure 4A and 4B). Importantly, co-treatment with these inhibitors markedly reversed the acquisition of mesenchymal cell markers upon ascites treatment (Figure 4C and Supplementary Figure S4A) and also reversed the ascites induced JAK2 and STAT3 phosphorylation (Figure 4D and Supplementary Figure S4B). These results suggest that ascites activate JAK2-STAT3 signaling pathway via IL-6 and increase SKOV-3 cell invasion.

### Ascites increase invasion only in ovarian cancer cells with IL-6R expression on cell membrane

**Figure 5:**
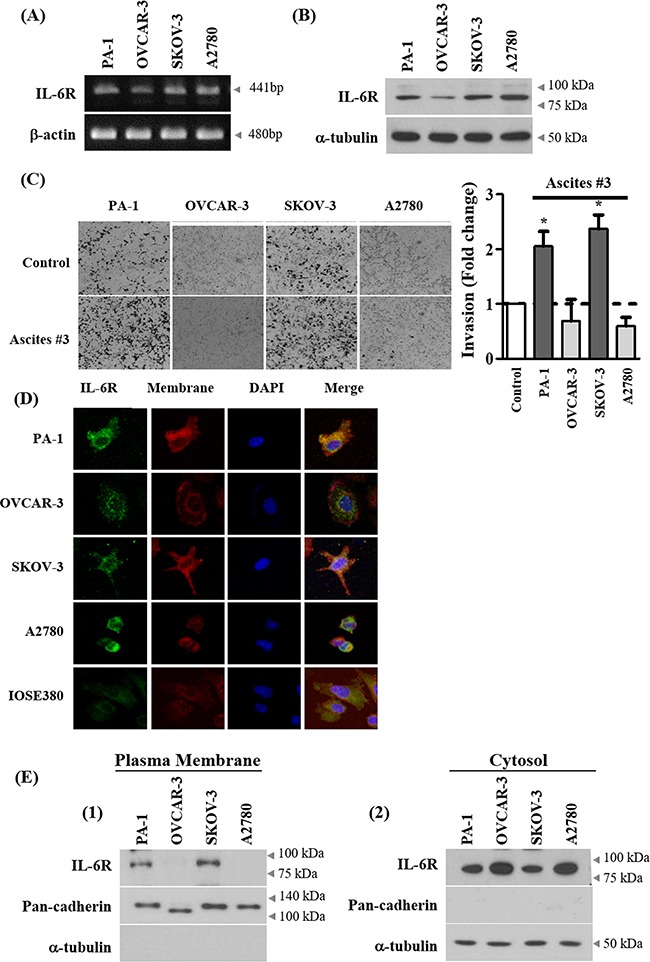
Expression of IL-6R on cell membrane correlates with ascites-induced invasion **A.** Basal mRNA expression of IL-6 receptor (IL-6R) in ovarian cancer cell lines were examined by RT-PCR. **B.** Basal protein expression of IL-6R in ovarian cancer cell lines was analyzed by western blot. α-tubulin was used as an internal control. **C.** Ovarian cancer cells were seeded into the upper chamber of Matrigel-coated membrane in transwells. Cell invasion were induced by ascites. After 24 hr, invaded cells at the bottom of the transwell were stained with 0.5% crystal violet and counted under a light microscope (magnification x200). **D.** Expression of IL-6R on cell membrane of ovarian cancer cells were examined by immunocytochemistry. The representative confocal microscopy figure (Green, IL-6R; red, plasma membrane; blue, DAPI) are shown. Original magnification 400x for all panels. **E.** Expression of IL-6R on cell membrane of ovarian cancer cell lines were examined by isolation of proteins from the plasma membrane and the cytosol fraction. The expression level of IL-6R was examined by western blot. Pan-cadherin and α-tubulin were used as an internal control. *, ** and *** represent *P* < 0.05, *P* < 0.01 and *P* < 0.001, respectively.

**Figure 6:**
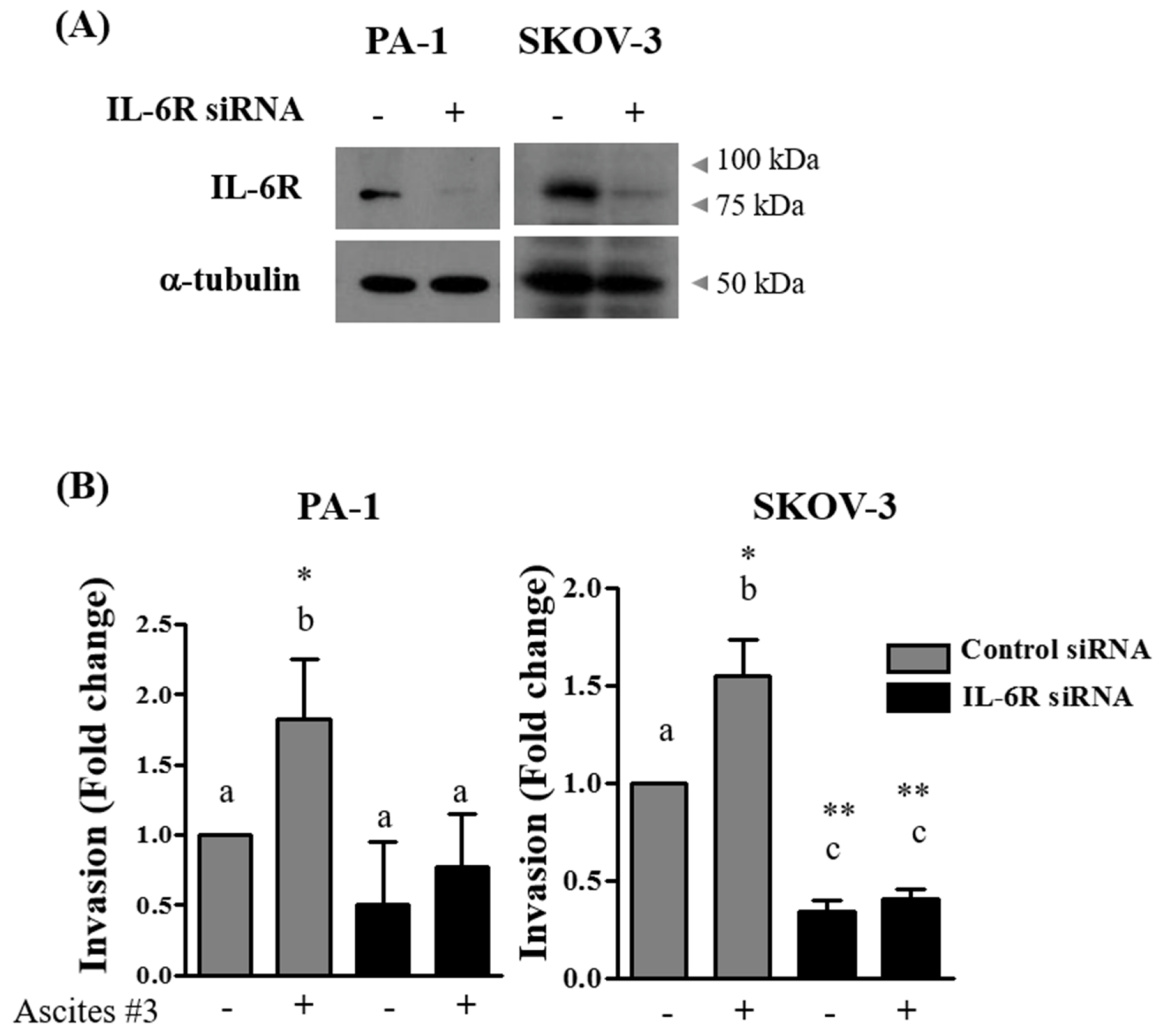
Silencing of IL-6R impairs ascites-induced invasion **A.** PA-1 and SKOV-3 cells were transfected with IL-6R targeted siRNA (100 nM) or scrambled RNA (100 nM) as a negative control. Silencing of IL-6R expression was examined by western blot. **B.** PA-1 and SKOV-3 cells were transfected with IL-6R targeted siRNA (100 nM) or negative control scrambled RNA (100 nM) for 24 hr. Cells were seeded into the upper chamber of Matrigel-coated membrane in transwells. Cell invasion were induced by 10% ascites. After 24, invaded cells at the bottom of the transwell were stained with 0.5% crystal violet and were counted under a light microscope (magnification x200). The data summarized in the bar charts are presented as mean ± SD of three independent fields. * and ** represent *P* < 0.05 and *P* < 0.01, respectively.

To determine whether ascites increase invasion in ovarian cancer cells in general or in a selective subset, additional ovarian cancer cell lines were tested. Four ovarian cancer cell lines including SKOV-3 were tested for expression of IL-6R at mRNA and protein levels. IL-6R were expressed in the 4 cell lines at both mRNA and protein levels (Figure 5A–5B and Supplementary Figure S5A–S5B). We found that ascites increase invasion only in PA-1 and SKOV-3 cells, but had no significant effect on OVCAR-3 and A2780 cell lines (Figure 5C). Interestingly, the effect of ascites treatment was not associated with endogenous IL-6 mRNA levels in ovarian cancer cell lines (Supplementary Figure S5C). Although the IL-6R protein is expressed in all four ovarian cancer cells tested, the expression of IL-6R on plasma membrane but not in cytosol of PA-1 and SKOV-3 cells correlate with ascites induced invasion (Figure 5D–5E and Supplementary Figure S5D). Finally, to link the IL-6R expression is critical for ascites mediated increased invasion in PA-1 and SKOV-3 cells, IL-6R was silenced and this process suppressed the ascites mediated enhanced invasion in these two cell lines (Figure 6A and 6B). These results demonstrate that IL-6 in ascites increase invasion via IL-6R on cell membrane and thus increase invasive properties only in a selective subset of ovarian cancer cells.

## DISCUSSION

Although the presence of ascites correlates with a poor prognosis in ovarian cancer patients, underlying molecular mechanism that lead to disease progression is poorly defined. Interestingly, the ascites presented in EOC patients constitute a dynamic reservoir of both pro-tumorigenic and anti-tumorigenic factors [20]. Among several factors accumulated in ascites, we found that IL-6 is enriched in ascites and is responsible for increased invasion, suggesting that suppressing its downstream signals might decrease migration and invasion.

Here we screened 36 pro-inflammatory cytokines and found IL-6 expression only in ovarian cancer patient derived ascites but absent in peritoneal fluids from benign condition (Figure 2A). Early studies have also reported enrichment of pro-inflammatory cytokines such as IL-6, CXCL10, and CD54 in ascites and have a pro-metastatic role in ovarian cancer progression [7, 20–23]. IL-6 signals through binding to heterodimer of IL-6R and gp130 [24, 25] and is associated with the metastatic phenotype in a range of cancers [12], and Lane et al. reported that IL-6 levels above 3 ng/ml in ascites is associated with poor prognosis in patients with ovarian cancer [10]. In line with this previous report, ascites collected in our study setting had higher IL-6 levels compared to reports by Lane et al. (Supplementary Figure S2C). Interestingly, early studies suggest that ascites in ovarian cancer patients forms a protective TME against drug-induced apoptosis by inducing survival signaling pathways such as PI3K/Akt [26, 27]. Moreover, the extent to which ascites served as protective TME was dependent on patients [9], which highlights inter-patient variability in the components of ascites. Similarly, although all three ascites tested in study increased invasion, we also observed that the magnitude of increased migration and invasion by ascites were variable among the patients (Figure 1A and 1B).

The metastasis is a critical step in determining the outcome of ovarian cancer patients, therefore preventing metastasis would improve prognosis in ovarian cancer patients [28]. Although a number of biological molecules related to metastasis in ovarian tumors have been elucidated [29], the precise role of IL-6R expression on cell membrane is undefined. Accumulating evidence suggests a rationale for anti- IL-6/IL-6R therapy for ovarian cancer treatment [30–32]. The inhibition of IL-6R using tocilizumab almost completely inhibited invasion promoted by the microenvironment [30]. Similarly, neutralization of IL-6 significantly enhanced the therapeutic efficacy of paclitaxel in mouse models of EOC by reducing tumor growth [33].

Our results demonstrate that IL-6R protein expression on plasma membrane of PA-1 and SKOV-3 cancer cells but not in cytosol predicts responsiveness to ascites in cell-based models of EOC invasion (Figure 5E1-2). Further study across a broader spectrum of patient-derived ascites and tumor samples is needed to fully investigate this hypothesis.

Collectively, the findings presented herein demonstrate that IL-6 in ascites function through membrane-bound IL-6R expressed in cancer cells and increase the EOC cell invasion via JAK2-STAT3 signaling. Targeting IL-6, IL-6R or JAK2/STAT3 may offer an efficient management of ascites induced migration and invasion in ovarian cancer patients.

## MATERIALS AND METHODS

### Cell culture, clinical samples and reagents

Human ovarian cancer cell lines, PA-1, OVCAR-3, and SKOV-3 used in this study were obtained from the American Type Culture Collection (Rockville, MD). A2780 was kindly gifted by Prof. Benjamin K. Tsang. With the exception of PA-1, these cell lines were grown in RPMI1640 (WelGENE, Seoul, Korea). PA-1 was cultured in MEM (WelGENE, Seoul Korea).

Induced ovarian surface epithelial cell line IOSE380 used in this study was kindly gifted by Prof. Young Kee Shin. The IOSE380 cell line was maintained in MCDB105:M199, 1:1 mixture. All culture media were supplemented with 10% FBS (Gibco-BRL, Gaithersberg, MD), and 100 μg/mL penicillin-streptomycin (P/S) (Invitrogen, Carlsbard, CA).

Ascites from three serous ovarian cancer patients and peritoneal fluid from two benign conditions were collected at the time of clinical intervention at the Seoul National University Hospital (Seoul, Korea). This study was approved by the Institutional Review Board (IRB) at Seoul national University Hospital (Registration number: 1409-1540-616), and prior written and informed consent was obtained from every patient. Ascites were centrifuged at 2500 rpm for 20 minutes. The acellular fractions were filtered (70 μm), aliquoted and stored at −80°C to minimize freeze-thaw.

### Wound healing assay

Cells were full plated with complete media in 6-well plates and incubated at 37°C for 24 hr. The complete medium was changed to either fresh complete media or 10% of the indicated ascites. The cell layer was scratched with a pipette tip to create an artificial wound. The wound closed area (in arbitrary unit) was measured using ImageJ software.

### Invasion assay

To assess the influence of ascites on the invasion, the Boyden chamber assay (transparent PET membrane with 8 μm pore size, BD Biosciences) was used. Inserts were pre-coated with growth factor reduced Matrigel (30 μg/insert; BD Biosciences). Cells were serum starved overnight and 40,000 cells were loaded to the upper chamber per insert and the inserts were placed to 10% of the indicated ascites or control medium. After 24 hr, cells were washed with PBS for three times and fixed with 4% formaldehyde for 1 hr at room temperature and stained with 0.5% crystal violet. Non-invaded cells were removed from the top filter surface with a cotton swab and invaded cells were viewed and photographed under a microscope. The invaded cells were counted using ImageJ software.

### Western blotting

Protein lysates were prepared as described previously [34]. In brief, after cell extraction, proteins were separated by SDS/PAGE (6-15% gel, depending on specific protein assessed) followed by electrotransfer onto nitrocellulose membranes and probed with the indicated antibodies.

### Reagents and antibodies

WP1066 and TG101348 (Selleckchem) were used in this study. Anti-recombinant human interleukin-6 (anti-rh IL-6), rh interleukin-6 (rh IL-6) and normal goat IgG were purchased from R&D Systems (R&D Systems, MN). JAK2, pJAK2 (Y1007), STAT3, pSTAT3 (Y705) and N-cadherin were purchased from Cell signaling (Danvers, MA). Snail, E-cadherin, Vimentin, GAPDH and IL-6R were purchased from Santa Cruz Biotechnology (Santa Cruz, CA). α-tubulin antibody was obtained from Sigma-Aldrich. Alexa Fluor conjugated anti-rabbit antibody was obtained from Invitrogen (Carlsbad, CA).

### Ascites analysis using proteome profiler cytokine array

Measured by the Human Cytokine Array Kit (R&D Systems), according to the manufacturing protocols. Ascites from two ovarian cancer patients and peritoneal fluid from two benign conditions were subjected to human cytokine array to detect the relative expression levels of 36 different cytokines.

### Determination of IL-6 concentration by ELISA

IL-6 levels in ascites samples were determined by ELISA using the commercially available human Quantikine HS ELISA Kit (R&D Systems, MN). The assays were performed in duplicate according to the manufacturer's protocols. The detection threshold was 0.156 pg/ml. A quantity of 50 μl of undiluted ascites was added in each well.

### Depletion of soluble interleukin-6

10% ascites diluted in complete medium were incubated with 50 ng/ml of a goat polyclonal anti-rh interleukin-6 (anti-rh IL-6; R&D Systems, MN) or a normal goat IgG control (R&D Systems, MN) at 37°C for 6 hr. The media were used in invasion assay as described above.

### RT-PCR

Total RNA was extracted with Trizol reagent (Life Technologies, Gaithersburg, MD). Single-stranded cDNA was constructed by PrimeScript Reverse Transcriptase (Takara, Japan). PCR was performed with specific primers IL-6R sense 5′-TCC ACC CCC ATG CAG GCA CT-3′, antisense 5′-GTGCCACCCAGCCAGCTATC-3′(size, 441 bp); IL-6 sense 5′-TAG CCG CCC CAC ACA GAC AG-3′, antisense 5′-GGC TGG CAT TTG TGG TTG GG-3′ (size, 408 bp); and β–actin sense 5′-ACA CTG CCA TCT ACG AGC-3′, antisense 5′-AGG GGC CGG ACT CGT CAT ACT-3′ (size, 480 bp) using the following amplification conditions: denaturation (95°C, 30s), annealing (60°C, 30s), and extension (72°C, 1min) followed by 35 cycles (72°C, 10min).

### Small interfering RNA transfection

The siRNA-targeting IL-6R (100 nM) gene corresponds to sequence was CGA CUC UGG AAA CUA UUC ATT and scrambled RNA (100 nM) was used as a negative control (mBioTech, Gyeonggido, Korea). All cancer cells were transfected with siRNA oligonucleotides with Lipofectamine® 2000 (Life Technologies). At 24 hr post-transfection cells were harvested and subsequently used for invasion assay.

### Immunofluorescence microscopy

Cells were plated onto a cover-slip and grown overnight in normal cell culture condition. The cells were fixed with 4% formaldehyde and blocked with 5% of goat serum. The anti-IL-6R antibody (1:500), Alexa Fluor conjugated anti-rabbit antibody (1:1000) and the membrane specific dye (CellMask™ Deep Red Plasma Membrane Stain, Invitrogen) were used for IL-6R membrane localization. Imaging was performed using a Confocal-A1 imaging system (Nikon). Original magnification was 400x for all panels, respectively.

### Statistical analysis

Data were presented as mean ± SD of triplicate experiments. One-way ANOVA and, when appropriate, Student's t-test were used for statistical analyses. Significant difference among experimental groups was analyzed by Scheffe's post hoc test. All analyses were conducted using IBM SPSS statistics 21 (SPSS Inc., Chicago, IL). P values of < 0.05 were considered statistically significant.

## SUPPLEMENTARY FIGURES


