# Supplementary material for: Malignant ascites enhances migratory and invasive properties of ovarian cancer cells with membrane bound IL-6R in vitro
Source: Oncotarget. 2016 Nov 4;7(50):83148–59. doi: 10.18632/oncotarget.13074 (PMC5347759; doi:10.18632/oncotarget.13074)
Supplement: Supplementary file 1 [file oncotarget-07-83148-s001.pdf]

Malignant ascites enhances migratory and invasive properties of ovarian cancer cells with membrane bound IL-6R *in vitro*

SUPPLEMENTARY FIGURES

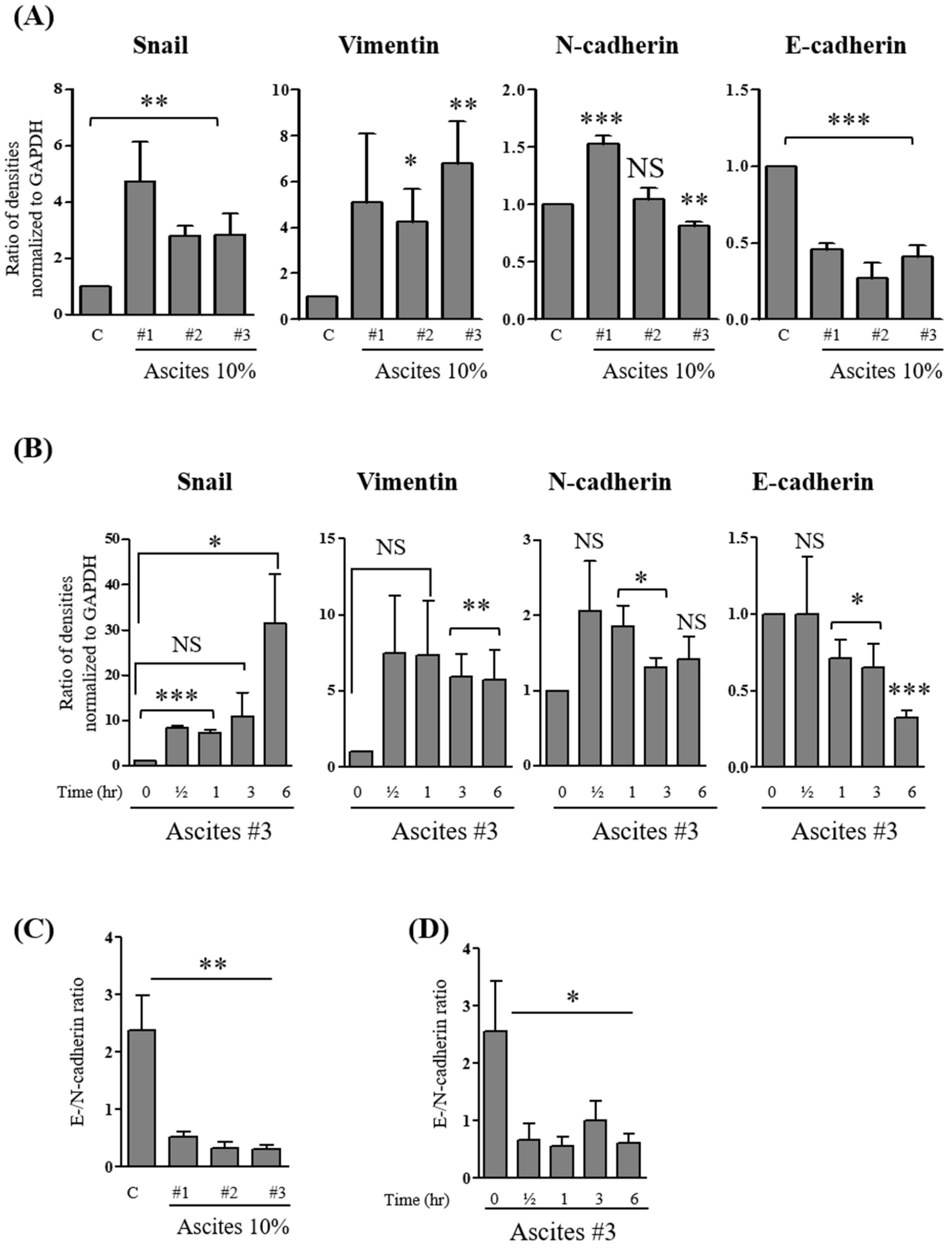

Supplementary Figure S1: Statistical analysis of EMT related protein expression, related to Figure 1. A. Statistical analysis of quantification of western blot in Figure 1D. EMT related proteins levels were quantitated by densitometry and normalized to GAPDH. Graph represents the fold induction upon treatment of ovarian cancer patient derived ascites collected from three patients, compared to complete media. **B.** Statistical analysis of quantification of western blot in Figure 1E. EMT related protein levels were quantitated as above. Graph represents the fold-induction upon treatment of ascites for 0 to 6 hr. **C-D.** Statistical analysis of quantification of N-cadherin protein densitometry normalized to E-cadherin in S1A and S1B. Graph represents the fold-induction upon treatment of ascites. SD is calculated from the mean of three independent experiments. *, ** and *** represent $P < 0.05$, $P < 0.01$ and $P < 0.001$, respectively.

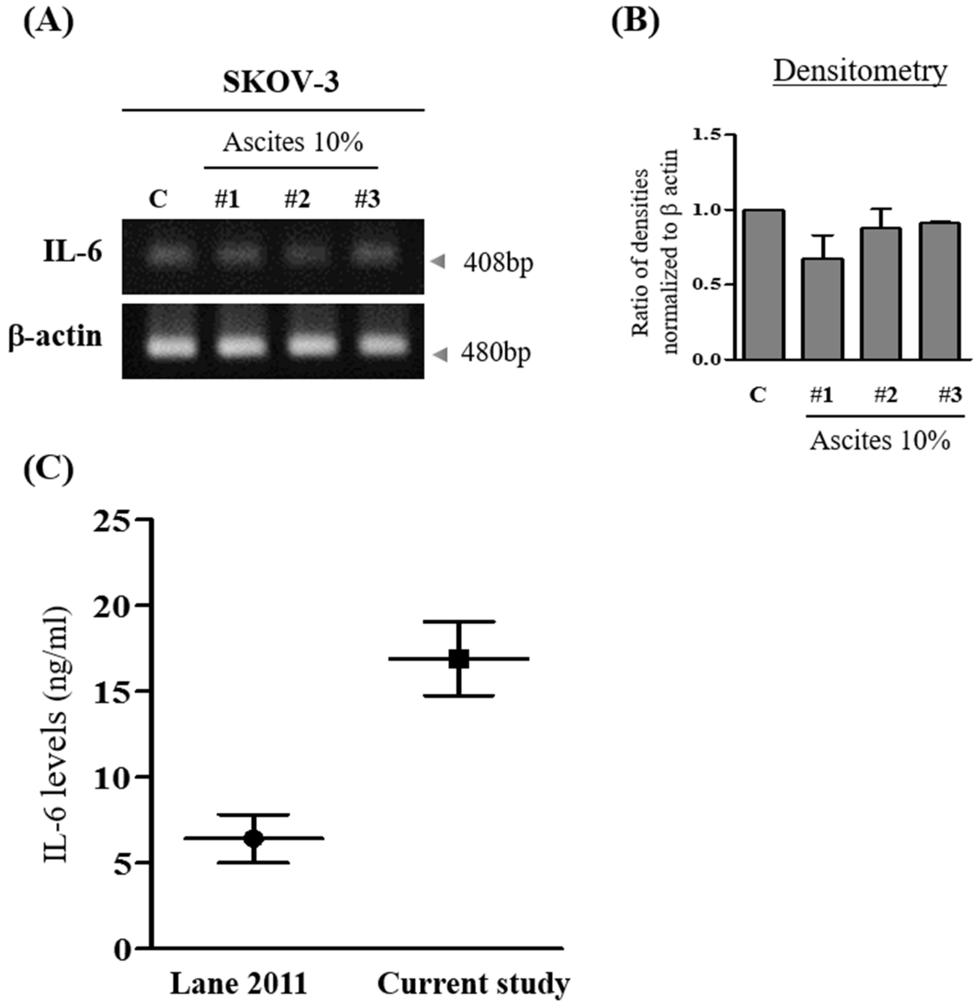

Supplementary Figure S2: Effect of ovarian cancer patient derived ascites on IL-6 expression in SKOV-3, related to Figure 2. A. Expression of IL-6 in SKOV-3 cells were examined by RT-PCR upon treatment of ovarian cancer patient derived ascites collected from three patients, compared to complete media. **B.** The quantification of RT-PCR in S2A. IL-6 expression levels were quantitated by densitometry and normalized to β actin. **C.** The concentration of IL-6 was determined by ELISA in ovarian cancer patient derived ascites (n = 34) compared to Lane et al. 2011 [10] Horizontal bar represents median value.

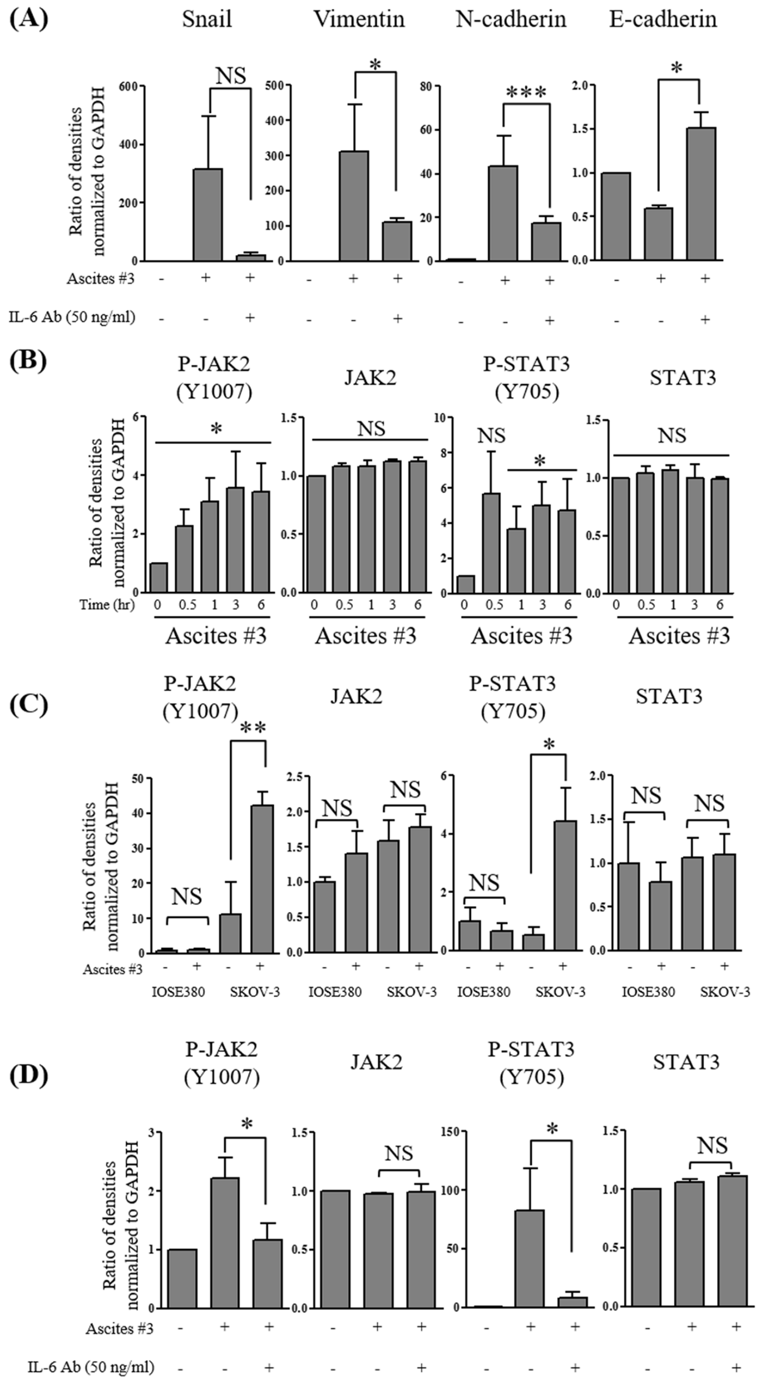

Supplementary Figure S3: Statistical analysis of EMT related protein expression and JAK2-STAT3 signaling, related to Figure 3. A. Statistical analysis of quantification of western blot in Figure 3C. EMT related proteins level were quantitated by densitometry and normalized to GAPDH. Graph represents the fold induction upon treatment of ascites with or without neutralizing IL-6 antibody. **B.** Statistical analysis of quantification of western blot in Figure 3D. JAK2-STAT3 phosphorylation were quantitated by densitometry and normalized to GAPDH. Graph represents the fold induction upon treatment of ovarian cancer patient derived ascites for 0 to 6 hr. **C.** Statistical analysis of quantification of western blot in Figure 3E. JAK2-STAT3 phosphorylation were quantitated by densitometry as above. Graph represents the fold induction upon treatment of ovarian cancer patient derived ascites for 0.5 hr in IOSE380 and SKOV-3. **D.** Statistical analysis of quantification of western blot in Figure 3F. JAK2-STAT phosphorylation were quantitated by densitometry as above. Graph represents the fold induction upon treatment of ascites with or without neutralizing IL-6 antibody. SD is calculated from the mean of three independent experiments. *, ** and *** represent $P < 0.05$, $P < 0.01$ and $P < 0.001$, respectively.

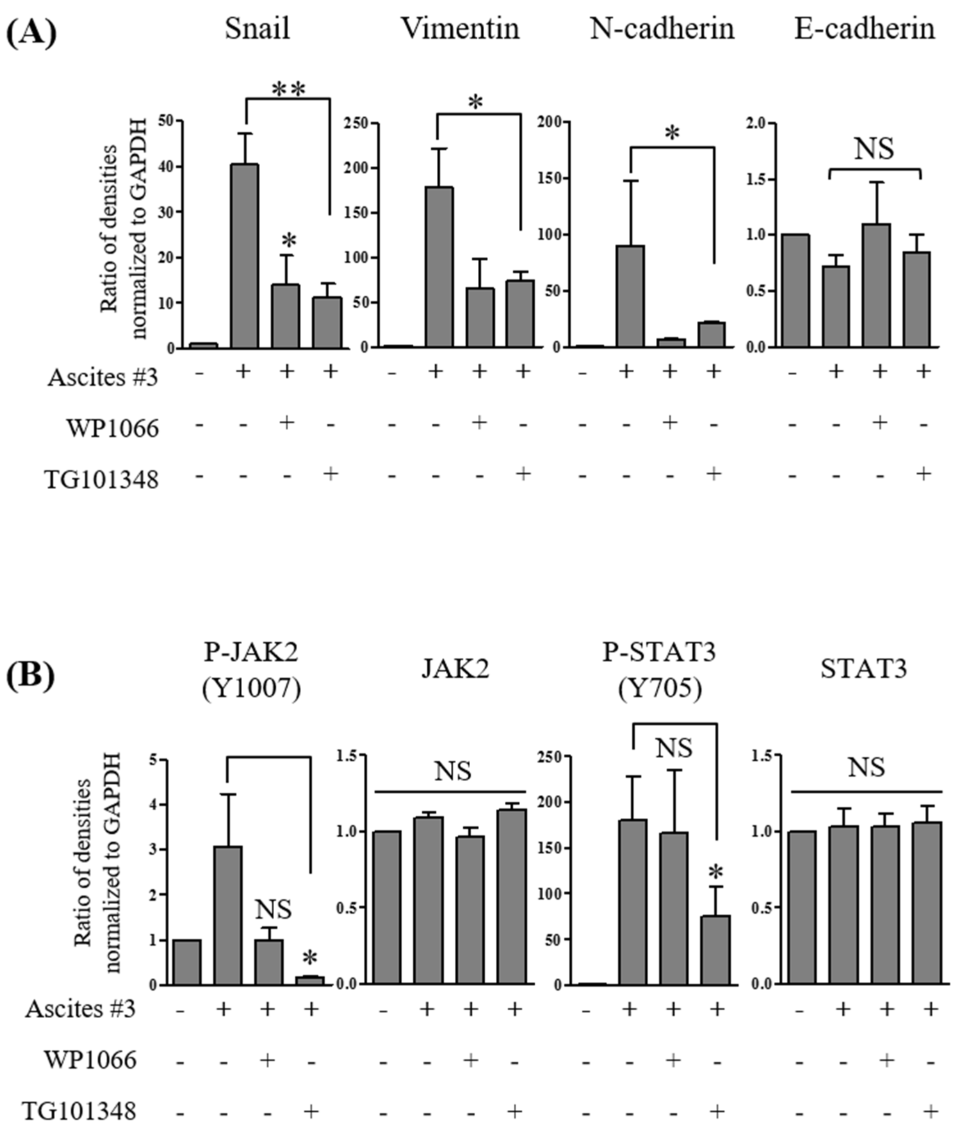

Supplementary Figure S4: Statistical analysis of EMT related protein expression and JAK2-STAT3 signaling, related to Figure 4. A. Statistical analysis of quantification of western blot in Figure 4C. EMT related proteins level were quantitated by densitometry and normalized to GAPDH. Graph represents the fold induction upon treatment of ascites with or without JAK2 and STAT3 inhibitors. **B.** Statistical analysis of quantification of western blot in Figure 4D. JAK2-STAT3 phosphorylation were quantitated by densitometry and normalized to GAPDH. Graph represents the fold induction upon treatment of ovarian cancer patient derived ascites with or without JAK2 and STAT3 inhibitors. SD is calculated from the mean of three independent experiments. * and ** represent $P < 0.05$ and $P < 0.01$, respectively.

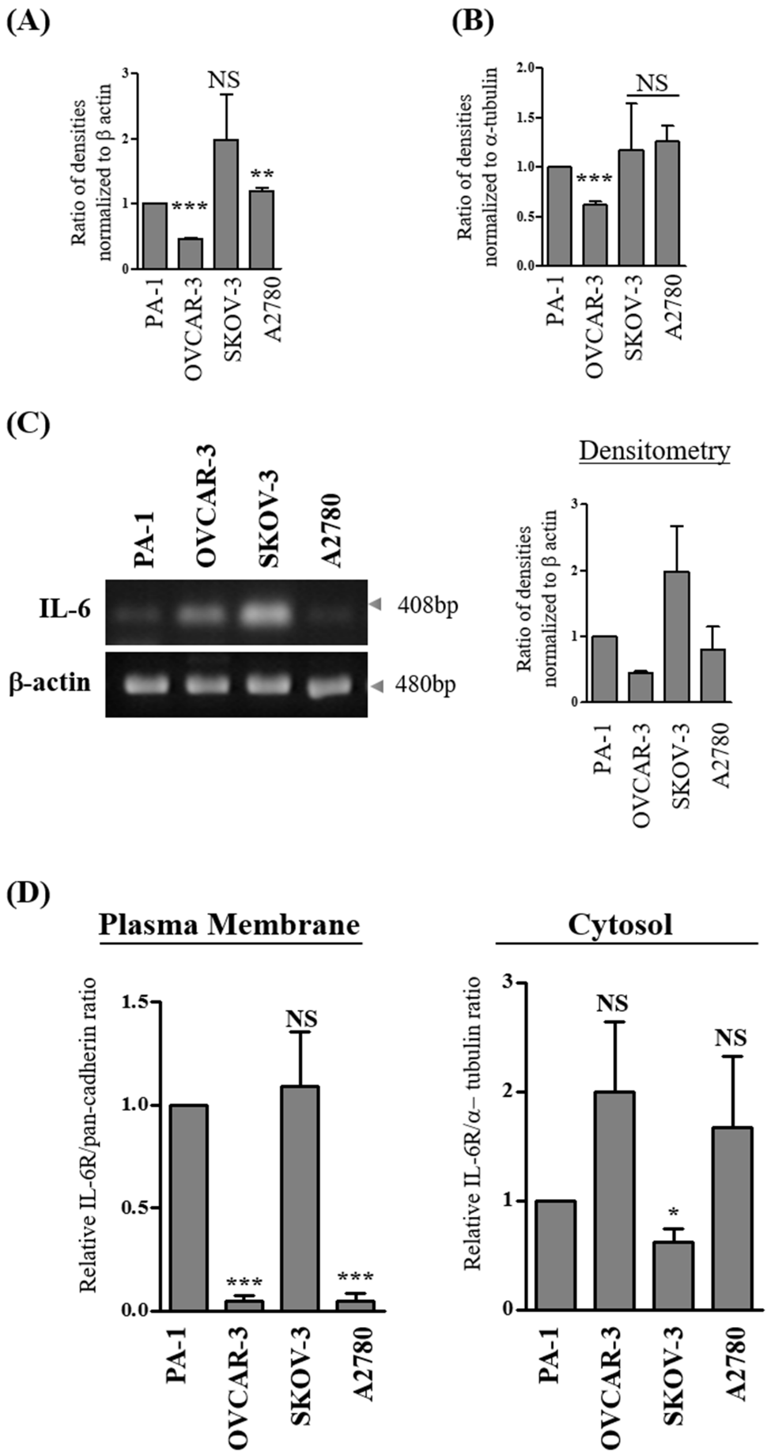

Supplementary Figure S5: IL-6 and IL-6R expression in ovarian cancer cell lines, related to Figure 5. A. Statistical analysis of quantitation of RT-PCR in Figure 5A. IL-6R mRNA expression levels were quantitated by densitometry and normalized to β actin. SD is calculated from the mean of three independent experiments. **B.** Statistical analysis of quantitation of western blot in Figure 5B. IL-6R expression levels were quantitated by densitometry and normalized to α tubulin. SD is calculated from the mean of three independent experiments. **C.** Expression of IL-6 in ovarian cancer cell lines were examined by RT-PCR. The representative figure and quantified data from three independent experiments were shown. **D.** Statistical analysis of quantitation of western blot in Figure 5E. SD is calculated from the mean of three independent experiments. ** and *** represent $P < 0.01$ and $P < 0.001$, respectively.
